# Clinical Characteristics, Viral Etiology, Management, and Prognosis of Pediatric Fulminant Myocarditis: A 10-Year Single-Center Study

**DOI:** 10.3390/v18091004

**Published:** 2026-09-12

**Authors:** Qingli Yang, Lijing Ye, Liting Wang, Yi Zhang, Yi Zhu, Jun Shen

**Affiliations:** 1 Department of Infectious Diseases, National Children’s Medical Center, Children’s Hospital of Fudan University, Shanghai 201102, China; 25211240019@m.fudan.edu.cn (Q.Y.); meian_2004@163.com (L.Y.); 24211240020@m.fudan.edu.cn (L.W.); 23211240031@m.fudan.edu.cn (Y.Z.); 2 Department of Clinical Epidemiology and Clinical Trial Unit, National Children’s Medical Center, Children’s Hospital of Fudan University, Shanghai 201102, China; zhangyi_chfd@fudan.edu.cn; 3 Pediatric Department, Children’s Hospital of Fudan University at Qidong, Nantong 226200, China

**Keywords:** fulminant myocarditis, viral myocarditis, pediatrics, viral etiology, extracorporeal membrane oxygenation, outcomes

## Abstract

**Background:** Fulminant myocarditis (FM) is a life-threatening inflammatory cardiac disease in children, frequently triggered by viral infections. Although mechanical circulatory support has improved short-term survival, data on viral etiology and long-term outcomes remain limited, particularly in Asian populations. **Methods:** We conducted a mixed retrospective–prospective analysis of pediatric FM patients at the Children’s Hospital of Fudan University from 2015 to 2025. Clinical data from 2015 to 2023 were collected retrospectively, while data from 2024 onward were collected prospectively. Clinical data, microbiological findings (PCR and metagenomic next-generation sequencing), treatment strategies, and outcomes were extracted from electronic medical records. Follow-up data for survivors were collected through December 2025. **Results:** A total of 53 children with FM were included. Median age was 91.5 months; 43.4% were male, and 56.6% were female. Common presenting symptoms included fever (64.2%) and vomiting (56.6%). Microbiological evidence was identified in 11 patients (20.8%), with rhinovirus (5.7%) and influenza virus (5.7%) being the most frequent, followed by enterovirus (3.8%) in peripheral specimens. During hospitalization, 79.2% required mechanical ventilation, 64.2% received extracorporeal membrane oxygenation (ECMO), and 45.3% underwent continuous renal replacement therapy (CRRT). Intravenous immunoglobulin (IVIG) was administered to 84.9%. In-hospital mortality was 13.2% (7/53), and 17.0% (9/53) of patients were discharged against medical advice (DAMA). Among 35 followed patients (median 12.4 months), most achieved favorable cardiac recovery; however, persistent conduction abnormalities, structural cardiac changes, and neurological sequelae were observed in a minority. **Conclusions:** Pediatric FM carries substantial in-hospital morbidity and resource utilization, despite favorable recovery rates in most survivors. The low pathogen detection rate in peripheral blood and the lack of endomyocardial tissue sampling preclude definitive conclusions regarding the underlying etiology, whether active viral replication or immune-mediated injury predominates. Endomyocardial biopsy-based investigations are urgently needed to clarify the underlying pathobiology.

## 1. Introduction

Myocarditis is defined as an inflammatory disease of the myocardium, and its diagnosis can be established by clinical presentation in conjunction with cardiac magnetic resonance (CMR) or endomyocardial biopsy (EMB) findings, in accordance with the 2025 European Society of Cardiology (ESC) Guidelines for the management of myocarditis and pericarditis [1]. Fulminant myocarditis (FM) represents the most severe clinical phenotype of acute myocarditis in children, characterized by abrupt onset, profound ventricular dysfunction, life-threatening arrhythmias, and cardiogenic shock [1,2].

Although relatively uncommon, FM accounts for a substantial proportion of pediatric myocarditis-related deaths and may contribute to 10–20% of sudden unexplained deaths in children [3]. The pathogenesis of FM involves a complex interplay between direct viral injury and a dysregulated host immune response [1,2]. Consequently, early recognition and timely initiation of circulatory support remain critical determinants of favorable outcomes [1,2,3].

Viral infections are the leading cause of myocarditis across all age groups, but they account for the vast majority of cases in children, where nonviral causes are relatively rare [4,5]. RNA viruses, particularly enteroviruses (Coxsackievirus B and echoviruses), are the most frequent cause of pediatric viral myocarditis, especially in neonates and infants [5]. Among DNA viruses, adenoviruses are another major cause frequently identified in endomyocardial biopsies [6,7]. Parvovirus B19 has emerged as a relevant etiologic agent of pediatric myocarditis, particularly during recent epidemiological surges in Europe and the United States [8]. Human herpesvirus 6 (HHV-6) has been increasingly recognized in pediatric myocarditis cases and may play a role in both acute and chronic disease processes [5]. Influenza A and B viruses, though relatively uncommon causes of myocarditis, can lead to fulminant presentations requiring intensive circulatory support [9].

Current evidence suggests that immune-mediated myocardial injury, rather than direct pathogen-induced cytotoxicity alone, plays a major role in disease progression [1,4]. Pediatric-specific immune responses—including robust innate responses but limited immune memory—alter susceptibility to viral infection and recovery trajectories [5,10]. Consequently, current management mainly focuses on hemodynamic stabilization, immunomodulatory therapy, and advanced mechanical circulatory support [1,2].

Over the past decade, remarkable advances in pediatric critical care have substantially improved the prognosis of FM. The increasing availability of ECMO, mechanical ventilation, and CRRT has significantly increased survival [2,11,12]. Nevertheless, considerable heterogeneity remains among published studies regarding mortality and long-term prognosis [11,12]. Existing pediatric studies have mainly focused on in-hospital management, whereas data regarding long-term outcomes and viral etiological profiles remain limited, particularly in Asian pediatric populations [13,14,15,16].

Therefore, we conducted a retrospective, real-world cohort study of children diagnosed with FM at a tertiary pediatric hospital in China. The present study aimed to comprehensively describe demographic characteristics, presenting symptoms, microbiological findings, treatment strategies, in-hospital outcomes, and long-term follow-up results, with particular emphasis on viral etiology and its clinical implications.

## 2. Materials and Methods

### 2.1. Study Design and Patient Selection

This mixed retrospective–prospective single-center study included consecutive pediatric patients diagnosed with FM admitted to the Children’s Hospital of Fudan University between 1 January 2015 and 31 December 2025. Clinical data from 2015 to 2023 were collected retrospectively, while data from 2024 onward were collected prospectively. Patients younger than 18 years who fulfilled the diagnostic criteria for FM were eligible. Diagnosis was established according to contemporary international recommendations, including clinical presentation, laboratory findings, electrocardiographic abnormalities, echocardiographic findings, and disease course [1,2]. A total of 53 patients were enrolled, including 46 identified retrospectively from the period 2015–2023 and 7 enrolled prospectively from the period 2024–2025.

Patients aged <18 years were included if they met the contemporary international diagnostic criteria for fulminant myocarditis (FM), encompassing clinical presentation, laboratory tests, electrocardiographic (ECG) and echocardiographic findings, and disease course [1,2]. Complete medical records were required for enrollment.

Exclusion criteria comprised pre-existing congenital heart disease, primary cardiomyopathies (e.g., dilated or hypertrophic cardiomyopathy), other identifiable causes of cardiogenic shock (e.g., septic shock, acute coronary syndrome), or incomplete medical records that precluded definitive diagnosis.

### 2.2. Data Collection

Clinical data were independently extracted from electronic medical records by two investigators using a standardized case report form. Variables collected included demographic characteristics, time intervals from symptom onset to admission, presenting manifestations, laboratory findings, electrocardiographic and echocardiographic findings, microbiological investigation results, therapeutic interventions, in-hospital outcomes, and long-term follow-up information.

### 2.3. Variable Definitions

Presenting symptoms were defined as symptoms documented before or at first medical evaluation. Laboratory variables used the first available acute-phase results. Echocardiographic left ventricular ejection fraction (LVEF) was recorded when numerical values were available. Electrocardiographic abnormalities included atrioventricular block, bundle branch block, ventricular tachycardia, ventricular fibrillation, and ST-T abnormalities.

Microbiological investigations included respiratory specimens (nasopharyngeal swabs, sputum, and alveolar lavage fluids), stool and blood cultures, and metagenomic next-generation sequencing (mNGS). When clinically indicated, metagenomic next-generation sequencing (mNGS) was performed, which routinely covered DNA viruses, including parvovirus B19 and human herpesvirus 6 (HHV-6). Prior to 2020, NPS and other respiratory specimens were tested using a DFA assay (Chemicon Respiratory Virus Diagnostic Kit, Chemicon International Inc., Temecula, CA, USA); after 2020, the RespiFinder 2SMART multi-PCR assay (GeneoDx Biotech Co., Shanghai, China) was employed instead. Detailed methods have been described previously; DFA detects 7 respiratory viruses (RSV, FLU A/B, PIV1–3, ADV), while RespiFinder covers 21 respiratory pathogens with higher sensitivity [17,18]. A patient was considered to have microbiological evidence if any pathogen was identified through clinically indicated testing.

Therapeutic interventions were categorized into three groups: immunomodulatory therapy (IVIG and corticosteroids), organ support (mechanical ventilation, ECMO, and CRRT or blood purification), and cardiac devices (temporary or permanent pacemaker implantation).

Follow-up information was obtained from outpatient medical records. Cardiac recovery was classified as complete recovery (normalization of cardiac function), persistent cardiac abnormalities (reduced LVEF, ventricular remodeling, ventricular aneurysm, clinically significant valvular disease, or permanent conduction abnormalities requiring pacemaker), or neurological sequelae (developmental delay, hypoxic–ischemic brain injury, or persistent neurological dysfunction).

### 2.4. Study Outcomes

Primary outcomes included in-hospital mortality and recovery of cardiac function during follow-up. Secondary outcomes included the requirement for mechanical ventilation, ECMO, CRRT, pacemaker implantation, length of hospital stay, discharge status, and long-term cardiac and neurological outcomes.

### 2.5. Statistical Analysis

All statistical analyses were performed using R software (version 4.6.0; R Foundation for Statistical Computing, Vienna, Austria) and RStudio (Posit Software, PBC, 2022.07.0, Boston, MA, USA). Descriptive data are presented as medians (IQRs) for continuous variables and as frequencies (percentages) for categorical variables. The normality of continuous variables was assessed using the Shapiro–Wilk test. Given the non-normal distribution of most continuous variables, between-group comparisons were performed using the Mann–Whitney *U* test. Categorical variables were compared using Fisher’s exact test. All tests were two-sided, and a *p* value < 0.05 was considered statistically significant. Given the exploratory nature of the study and the limited sample size, no adjustment was made for multiple comparisons; all reported *p* values should be interpreted as descriptive rather than confirmatory.

## 3. Results

### 3.1. Baseline Characteristics and Clinical Presentation

A total of 53 children with FM were included, comprising 46 retrospective cases (2015–2023) and 7 prospective cases (2024–2025). The cohort had a median age of 91.5 months (IQR, 51.3–119.6), with 43.4% (23/53) males and 56.6% (30/53) females. The median interval from symptom onset to admission was 3 days (IQR, 2–5), and the median hospital stay was 26 days (IQR, 15–32).

**Figure 1:**
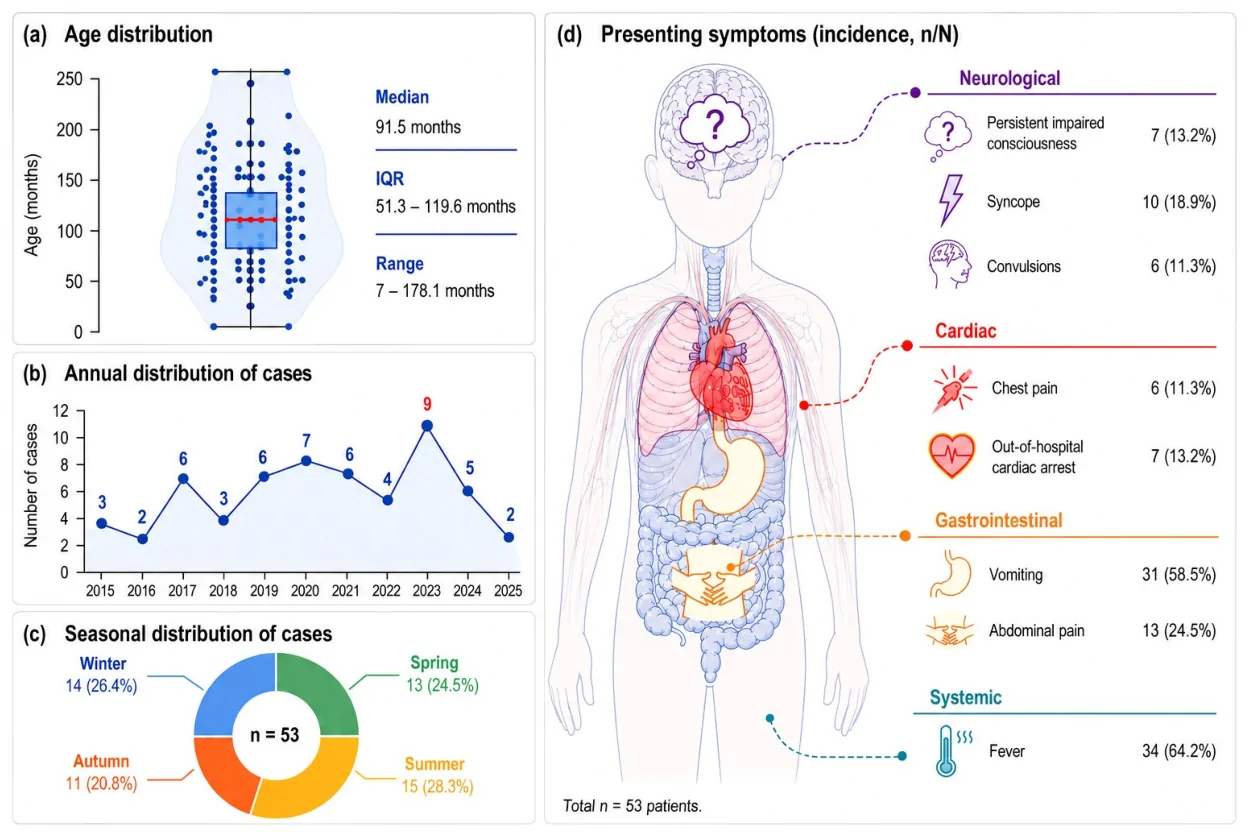
Demographic characteristics and clinical presentation: (**a**) age distribution, showing the highest frequency among school-aged children; (**b**) annual distribution from 2015 to 2025, without a consistent trend; (**c**) seasonal distribution, with cases occurring throughout all four seasons; (**d**) frequency of presenting symptoms, with fever (64.2%) and vomiting (56.6%) being most common.

Figure 1 illustrates demographic characteristics and clinical presentation. As shown in Figure 1a, cases were distributed across a wide pediatric age range, with the highest frequency among school-aged children. Figure 1b demonstrates that cases occurred throughout the study period without a consistent annual trend. The seasonal distribution in Figure 1c shows that cases occurred throughout all four seasons without obvious clustering. Regarding clinical presentation (Figure 1d), fever (64.2%, 34/53) and vomiting (56.6%, 30/53) were the most common presenting symptoms, followed by abdominal pain (24.5%, 13/53) and syncope (18.9%, 10/53). Chest pain, dyspnea, seizure, and disturbance of consciousness were less frequently reported.

### 3.2. Cardiac Examination and Microbiological Findings

Left ventricular systolic dysfunction was identified in the majority of patients during the acute phase. Ventricular wall thickening and pericardial effusion were observed in 18.9% and 22.6%, respectively. Electrocardiographic abnormalities were common, with atrioventricular conduction block (30.2%) and ventricular tachycardia (26.4%) being the most frequent findings (Table 1).

No endomyocardial biopsy specimens were obtained in this cohort. Microbiological investigations of blood, stool, and respiratory specimens identified pathogens in 11 patients (20.8%). Rhinovirus (5.7%, 3/53) and influenza virus (5.7%, 3/53) were the most frequently detected microorganisms, followed by enterovirus (3.8%, 2/53). Adenovirus, norovirus, and *Haemophilus influenzae* were each identified in one patient (1.9%) (Table 2). Among patients with PCR-confirmed viral detection, all positive results represented single-pathogen infections; no coexisting viral pathogens were identified in the same patient.

### 3.3. Treatment Strategies and In-Hospital Outcomes

**Figure 2:**
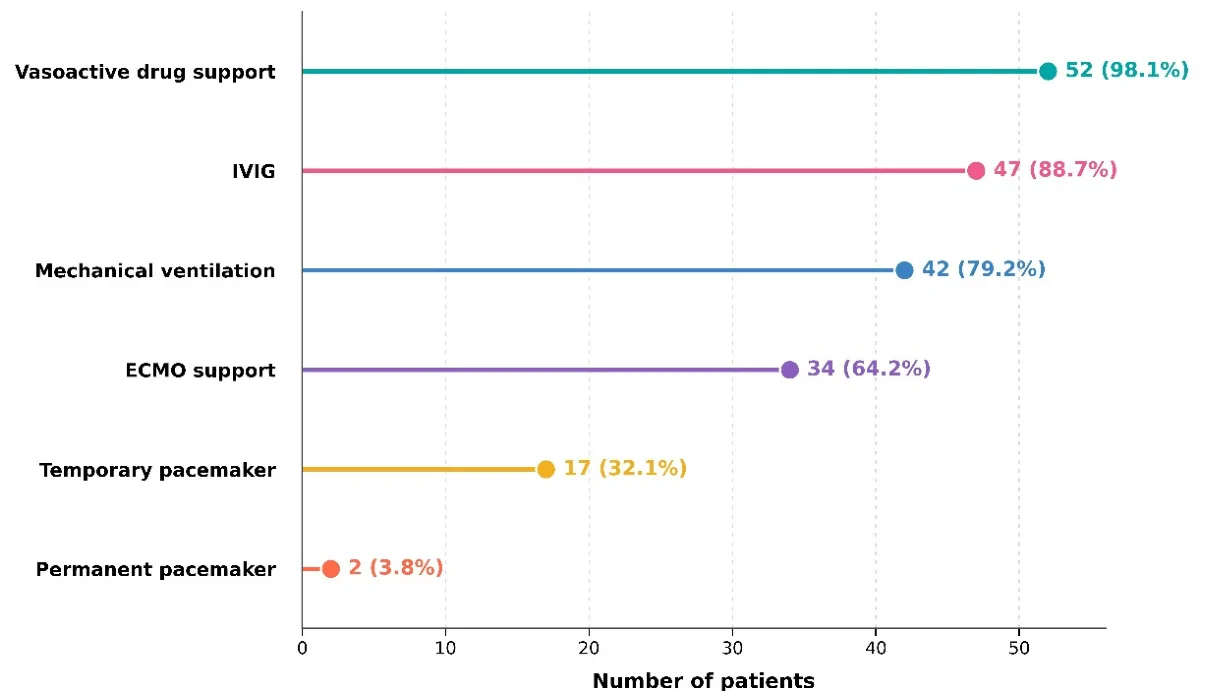
Treatment strategies and in-hospital outcomes. IVIG, intravenous immunoglobulin; ECMO, extracorporeal membrane oxygenation.

Figure 2 provides a comprehensive overview of treatment strategies and outcomes in this cohort. As shown in the figure, a high proportion of patients required intensive interventions. Mechanical ventilation was administered to 79.2% (42/53) of patients, reflecting the severity of respiratory and hemodynamic compromise. ECMO was employed in 64.2% (34/53) of cases, indicating the frequent need for advanced circulatory support in this critically ill population. CRRT was utilized in 45.3% (24/53) of patients, consistent with the high prevalence of multi-organ dysfunction. Temporary pacemaker implantation was required in 32.1% (17/53) of patients, highlighting the substantial burden of life-threatening conduction abnormalities during the acute phase. Immunomodulatory therapy was widely used, with IVIG administered to 84.9% (45/53) of patients and corticosteroids given to 66.0% (35/53). The use of vasoactive drugs was nearly universal (98.1%, 52/53).

Regarding in-hospital outcomes, 37 patients (69.8%) survived to hospital discharge, 7 patients (13.2%) died during hospitalization, and 9 patients (17.0%) were DAMA. As DAMA cases were not captured in the mortality endpoint, the observed in-hospital mortality rate of 13.2% may not reflect the full burden of fatal outcomes in this cohort.

Table 3 summarizes the clinical characteristics stratified by in-hospital outcome. Non-survivors had a significantly shorter hospital stay compared with survivors [2 (0.5–3.5) vs. 27 (19.5–33.5) days, *p* < 0.001]. Although non-survivors more frequently presented with vomiting (85.7% vs. 54.3%) and had higher rates of mechanical ventilation (100% vs. 76.1%) and ECMO support (85.7% vs. 60.9%), these differences did not reach statistical significance, likely due to the limited sample size. Demographic characteristics and most other presenting symptoms were generally comparable between the two groups (all *p* > 0.05).

Table 4 compares the clinical characteristics of patients with and without ECMO support. Not surprisingly, the ECMO group had significantly higher rates of mechanical ventilation (100% vs. 47.4%, *p* < 0.001) and vasoactive drug support (100% vs. 94.7%, *p* = 0.026), along with more frequent out-of-hospital cardiac arrest (20.6% vs. 0%, *p* = 0.037) and reduced left ventricular systolic function (76.5% vs. 57.9%, *p* = 0.157), indicating greater disease severity in patients requiring ECMO. In addition, male sex was significantly more prevalent in the ECMO group (58.8% vs. 15.8%, *p* = 0.002). No significant differences were observed between the two groups regarding age, most presenting symptoms, or length of hospital stay (all *p* > 0.05).

### 3.4. Long-Term Outcomes

**Figure 3:**
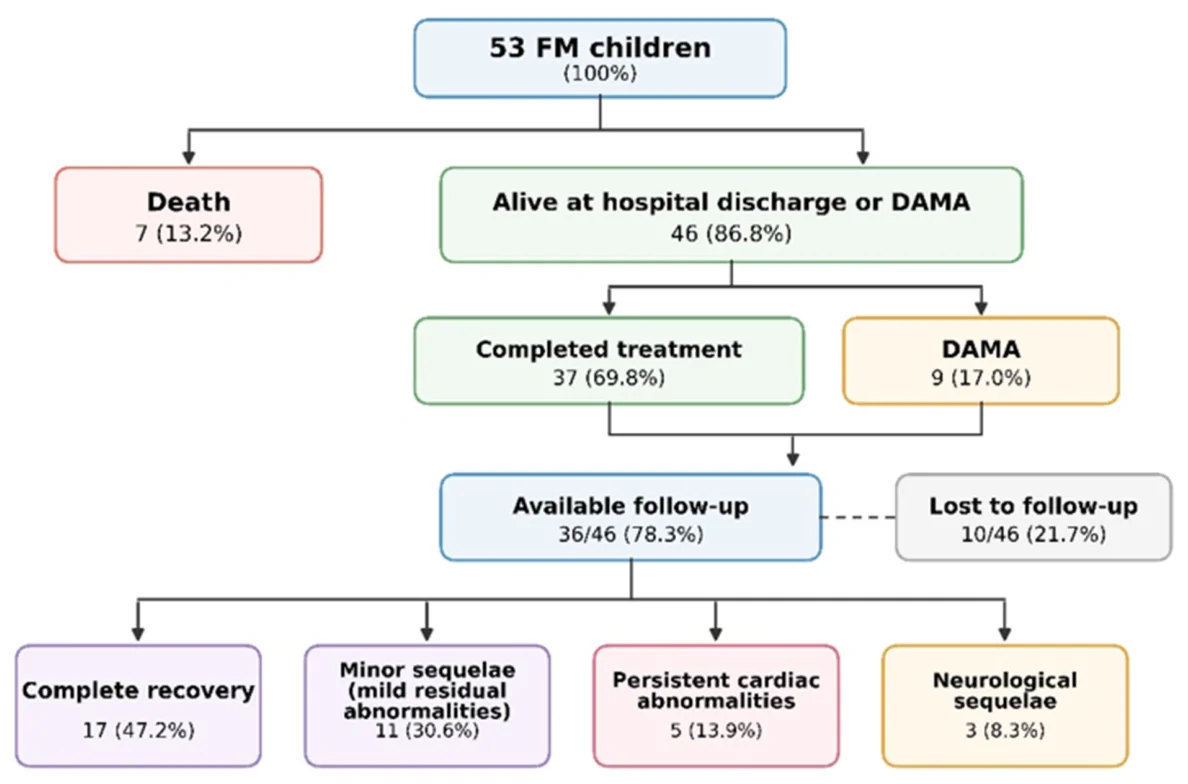
Flow diagram of clinical outcomes and follow-up status in 53 children with fulminant myocarditis. Most achieved complete recovery or minor residual abnormalities; a small proportion developed persistent cardiac abnormalities or neurological sequelae. FM, fulminant myocarditis; DAMA, discharged against medical advice.

Long-term follow-up data were available for 36 of 46 eligible patients (78.3%), with a median follow-up duration of 12.4 months (IQR, 2.5–34.1 months). Figure 3 illustrates follow-up outcomes. Most survivors demonstrated favorable recovery of cardiac function. Echocardiographic abnormalities resolved in the majority, whereas a small number exhibited persistent cardiac abnormalities, including reduced LVEF, ventricular enlargement, ventricular aneurysm, or clinically significant valvular disease.

Persistent electrocardiographic abnormalities were observed, most commonly atrioventricular conduction block and right bundle branch block. Two patients required permanent pacemaker implantation for persistent complete atrioventricular block.

Neurological sequelae were identified in 3 of the 36 survivors (8.3%). Persistent electrocardiographic abnormalities were observed in 5 patients (13.9%). Mild residual abnormalities such as minor electrocardiographic changes without functional impairment or well-controlled arrhythmias not requiring intervention were documented in 11 patients (30.6%). The remaining 17 survivors (47.2%) showed satisfactory clinical recovery without recurrent hospitalization attributable to FM.

Table 5 summarizes the baseline characteristics of patients stratified by long-term outcome. Overall, patients with residual sequelae and those with complete recovery were broadly comparable across most demographic and clinical variables. Length of hospital stay was significantly longer in patients with complete recovery [32 (30–39) vs. 26 (21–33.5) days, *p* = 0.018].

## 4. Discussion

In this 10-year retrospective cohort of 53 children with fulminant myocarditis, we comprehensively evaluated clinical characteristics, viral etiology, management strategies, and long-term prognosis. Several important findings emerged.

### 4.1. Disease Severity and Supportive Care

Pediatric FM in our cohort was associated with substantial disease severity, as reflected by the high proportions of patients requiring mechanical ventilation (79.2%), ECMO (60.4%), and CRRT (45.3%) (Figure 1). The in-hospital mortality of 13.2% (7/53) is comparable to recent multicenter data from China (16.6% in 187 children) and a single-center study (20% in 40 children) [15]. However, the 17.0% rate of DAMA warrants particular attention. This phenomenon, which is not uncommon in retrospective studies from certain regions [15], may lead to underestimation of true mortality and should be carefully considered when interpreting survival rates [16]. The significantly shorter hospital stay observed in non-survivors (median 2 days) compared with survivors (27 days) reflects the rapid disease progression and early mortality in the most severely affected patients (Table 3), consistent with the aggressive clinical course typically described in pediatric FM [3,11]. Patients requiring ECMO support demonstrated greater disease severity at baseline, with significantly higher rates of mechanical ventilation, vasoactive drug support, and out-of-hospital cardiac arrest, consistent with the expected clinical profile of this high-risk subgroup (Table 4), and with previous reports on the use of mechanical circulatory support in critically ill children with FM [11,12].

### 4.2. Viral Etiology: Insights and Implications

The overall pathogen detection rate in our cohort was 20.8%, with rhinovirus and influenza virus being the most frequently identified pathogens (Table 2). This finding is consistent with previous pediatric studies [4,5,19]. Nevertheless, several important caveats must be acknowledged.

First, the use of specimens and the lack of standardized sampling timing preclude any definitive distinction between active viral replication and post-viral immune-mediated injury. Second, the relatively low detection rate does not necessarily indicate a minor role for viral triggers; rather, it may reflect the fact that myocardial damage often persists through host immune dysregulation even after peripheral viral clearance. Indeed, emerging evidence from single-cell sequencing has highlighted the crucial roles of CD8+ T cell hyperactivation via the CXCL12/CXCR4 axis and NLRP3 inflammasome-mediated pyroptosis in sustaining myocardial injury [20]. These mechanistic insights, however, were derived from independent experimental studies; our clinical data do not directly address these pathways and should be interpreted as correlative observations from peripheral specimens.

In addition, several methodological factors may also contribute to the low detection rate and warrant acknowledgment. Diagnostic assays evolved during the study period: DFA was used before 2020, whereas the more sensitive RespiFinder 2SMART multiplex PCR was adopted thereafter [18,19]. Consequently, detection rates for pre-2020 cases are likely underestimated. Second, specimen types were predominantly respiratory and stool samples. This is relevant because HHV-6 is optimally detected in blood, cerebrospinal fluid, or pericardial fluid [21], while parvovirus B19 requires specific PCR testing that was not systematically performed (except in mNGS cases when clinically indicated). Moreover, the median sampling time, 3 days after symptom onset, may fall after the peak viremic phase, when immune-mediated injury begins to predominate.

### 4.3. Long-Term Outcomes: Recovery and Residual Sequelae

One of the major strengths of this study is the relatively comprehensive long-term follow-up (78.3% of survivors, median 12.4 months) (Figure 3). Our findings demonstrate that the long-term prognosis of survivors is generally favorable. Most patients showed normalization of cardiac function, supporting the concept that FM-associated myocardial dysfunction is largely reversible with adequate supportive therapy. However, recovery of LVEF should not be equated with complete cardiac recovery. Several patients exhibited persistent electrocardiographic abnormalities, including atrioventricular block and right bundle branch block, despite normalization of ventricular function. Two children required permanent pacemaker implantation for persistent complete atrioventricular block. Although conduction abnormalities are common during the acute phase, most resolve as inflammation subsides; persistent high-grade block requiring permanent pacing appears uncommon but represents an important long-term complication [1,2]. Neurological sequelae, including developmental delay and hypoxic–ischemic brain injury, were observed in several patients, largely secondary to prolonged circulatory collapse or cardiac arrest. Successful management should therefore emphasize early restoration of systemic perfusion to minimize irreversible neurological injury (Table 5).

These findings indicate that hospital discharge should not be considered the endpoint of clinical management. Structured long-term follow-up, including clinical assessment, electrocardiography, echocardiography, and individualized evaluation of rhythm disturbances and neurological function, is essential for early identification of late complications and optimization of long-term quality of life.

### 4.4. Therapeutic Considerations and Clinical Implications

IVIG was administered to 84.9% of patients in our cohort, reflecting widespread clinical practice. However, its therapeutic efficacy remains controversial [22]. Some observational studies suggest IVIG may attenuate myocardial inflammation, while others have failed to demonstrate significant reduction in mortality or improvement in long-term ventricular function. Current international guidelines do not recommend routine IVIG for all patients but consider its use reasonable in selected pediatric cases, particularly when immune-mediated myocardial injury is suspected [1,2]. The role of corticosteroids similarly remains uncertain. Given the observational nature of our study, we cannot draw causal inferences regarding treatment efficacy.

Our findings have several practical implications. First, the high utilization of ECMO (60.4%) and mechanical ventilation (79.2%) underscores the need for tertiary pediatric centers to have robust mechanical circulatory support capabilities. Second, the low pathogen detection rate highlights the need for standardized, comprehensive viral testing protocols. Third, the occurrence of residual cardiac and neurological sequelae in a minority of survivors emphasizes the importance of structured, multidisciplinary long-term follow-up. Finally, the phenomenon of DAMA deserves increased awareness, as it may obscure true mortality rates and hinder complete outcome assessment.

## 5. Strengths and Limitations

### 5.1. Strengths

First, this study represents one of the relatively large single-center pediatric FM cohorts in China, covering a 10-year period with consecutive enrollment. Second, comprehensive clinical information was collected, including detailed microbiological investigation results. Third, relatively long-term follow-up data were available for a substantial proportion of survivors, allowing evaluation of both cardiac recovery and late complications.

### 5.2. Limitations

We acknowledge several methodological limitations, with the most critical being the absence of myocardial tissue analysis. All virological testing was performed on peripheral specimens (respiratory, stool, or blood) rather than endomyocardial biopsy or autopsy tissue; consequently, we could not assess intramyocardial viral genomes, immune cell infiltration, or histopathological patterns, the reference standard for distinguishing active viral replication from post-viral immune-mediated injury. This precludes any mechanistic attribution of FM based on our data.

Additional limitations include the retrospective single-center design with 53 cases, which may limit generalizability and introduce selection bias; the observational nature, which prevents causal inference regarding interventions; and the variation in diagnostic assays over time (DFA before 2020 vs. multiplex PCR thereafter) as well as the lack of systematic testing for HHV-6 and parvovirus B19, which may have underestimated pathogen detection. Variable follow-up durations and patient attrition may have also affected long-term outcome estimates. In addition, the high rate of DAMA (17.0%) may have introduced selection bias in the follow-up cohort and potentially underestimated true mortality and long-term complication rates, as outcomes for these patients could not be ascertained.

Finally, given the exploratory nature and limited sample size (7 in-hospital deaths), we did not perform multivariate regression or multiple comparison corrections; thus, the subgroup comparisons in Table 3, Table 4 and Table 5 are descriptive and should be viewed as hypothesis-generating rather than confirmatory.

Notwithstanding these caveats, this study represents one of the few systematically followed pediatric FM cohorts and offers valuable real-world data on clinical presentation, therapeutic strategies, and medium-term outcomes in this rare but life-threatening disease.

## 6. Conclusions

FM remains a life-threatening pediatric emergency requiring intensive care and advanced mechanical circulatory support. Although most survivors achieve favorable cardiac recovery, a minority develop persistent conduction abnormalities, structural changes, or neurological sequelae, underscoring that LVEF recovery does not equate to complete recovery. Structured long-term follow-up with electrocardiographic, echocardiographic, and neurological assessments should be routinely performed to detect late complications and guide rehabilitation. Enhanced viral testing protocols and strategies to reduce DAMA are warranted in future studies.

## Figures and Tables

**Table 1:** Echocardiographic and electrocardiographic findings in 53 children with fulminant myocarditis.

Variable	n (%)
**Echocardiographic findings**	
Left ventricular systolic dysfunction (LVEF < 55%)	33 (62.3)
Ventricular wall thickening	10 (18.9)
Pericardial effusion	12 (22.6)
Moderate-to-severe valvular regurgitation	7 (13.2)
**Electrocardiographic findings**	
Atrioventricular conduction block	16 (30.2)
Right bundle branch block	7 (13.2)
Sinus tachycardia	12 (22.6)
Ventricular tachycardia	14 (26.4)
Ventricular fibrillation	1 (1.9)

**Table 2:** Pathogens detected, specimen types, and diagnostic methods in 53 pediatric fulminant myocarditis cases.

Pathogens	Positive Cases (n)	Positive Rates (%)	Specimens	Methods
Rhinovirus	3	5.66	Nasopharyngeal swab	RT-PCR
Influenza virus	3	5.66	Nasopharyngeal swab	RT-PCR or LAAT
Enterovirus	2	3.77	Blood and Stool	RT-PCR
Adenovirus	1	1.89	Nasopharyngeal swab	PCR
Norovirus	1	1.89	Stool	RT-PCR
*Haemophilus influenzae*	1	1.89	Blood and nasopharyngeal swab	mNGS

RT-PCR, reverse-transcription polymerase chain reaction; mNGS, metagenomic next-generation sequencing; LAAT, latex agglutination antibody test.

**Table 3:** Clinical characteristics of patients stratified by in-hospital outcome ([IQR], n [%]).

Variable	In-Hospital Death (n = 7)	Discharged Alive (n = 46)	*p* Value
**Demographics**			
**Sex, n (%)**			0.441
Male	4 (57.1)	19 (41.3)	
Female	3 (42.9)	27 (58.7)	
Age, months, median (IQR)	110.7 (64.7–123.8)	90.7 (51.7–119.2)	0.380
**Presenting symptoms, n (%)**			
Fever	4 (57.1)	30 (65.2)	0.686
Vomiting	6 (85.7)	25 (54.3)	0.127
Abdominal pain	1 (14.3)	12 (26.1)	0.501
**Supportive therapies, n (%)**			
Vasoactive drug support	7 (100)	45 (97.8)	0.690
Mechanical ventilation	7 (100)	35 (76.1)	0.155
ECMO support	6 (85.7)	28 (60.9)	0.203
Length of hospital stay, days, median (IQR)	2 (0.5–3.5)	27 (19.5–33.5)	<0.001

Fisher’s exact test was used for categorical variables due to the small sample size in the non-survivor group, and the Mann–Whitney *U* test was applied for continuous variables, which were not normally distributed.

**Table 4:** Clinical characteristics of patients stratified by ECMO support.

Variable	ECMO (n = 34)	Non-ECMO (n = 19)	*p* Value
**Demographics**			
**Sex, n (%)**			0.002
Male	20 (58.8)	3 (15.8)	
Female	14 (41.2)	16 (84.2)	
Age, months, median (IQR)	84.8 (47.6–126.0)	102.2 (69.3–117.8)	0.562
**Presenting symptoms, n (%)**			
Fever	20 (58.8)	14 (73.7)	0.278
Vomiting	21 (61.8)	10 (52.6)	0.514
Abdominal pain	11 (32.4)	2 (10.5)	0.073
**Supportive therapies, n (%)**			
Vasoactive drug support	34 (100)	18 (94.7)	0.026
Mechanical ventilation	34 (100)	8 (42.1)	<0.001
Reduced left ventricular systolic function	26 (76.5)	11 (57.9)	0.157
Length of hospital stay, days, median (IQR)	26.5 (9.0–38.3)	25 (16–30)	0.531

Data are presented as median (IQR) for continuous variables and n (%) for categorical variables. Categorical variables were compared using Fisher’s exact test and continuous variables using the Mann–Whitney *U* test. ECMO, extracorporeal membrane oxygenation; IQR, interquartile range.

**Table 5:** Long-term outcomes of 46 patients with available follow-up data.

Variable	Complete Recovery (n = 17/46)	Residual Sequelae (n = 19/46)	*p* Value
**Demographics**			
Male sex, n (%)	10 (58.8)	5 (26.3)	0.051
Age, months, median (IQR)	98.7 (62.9–134.9)	102.2 (66.3–114.8)	0.904
**Presenting symptoms, n (%)**			
Fever	13 (76.5)	12 (63.2)	0.389
Vomiting	8 (47.1)	8 (42.1)	0.764
Abdominal pain	3 (17.6)	6 (31.6)	0.337
**Supportive therapies, n (%)**			
Vasoactive drug support	17 (100)	18 (94.7)	0.472
Mechanical ventilation	14 (82.4)	14 (73.7)	0.533
ECMO support	10 (58.8)	11 (57.9)	0.954
Reduced LV systolic function, n (%)	11 (64.7)	14 (73.7)	0.559
Length of hospital stay, days, median (IQR)	32 (30–39)	26 (21–33.5)	0.018

Data are presented as median (IQR) for continuous variables and n (%) for categorical variables. Categorical variables were compared using Fisher’s exact test, and continuous variables using the Mann–Whitney *U* test. IQR, interquartile range.

## Data Availability

The data presented in this study are available upon reasonable request from the corresponding authors.
